# Scale and translation-invariance for novel objects in human vision

**DOI:** 10.1038/s41598-019-57261-6

**Published:** 2020-01-29

**Authors:** Yena Han, Gemma Roig, Gad Geiger, Tomaso Poggio

**Affiliations:** 1 Center for Brains, Minds and Machines, MIT, 77 Massachusetts Ave, Cambridge, MA 02139 United States of America; 2 Computer Science Department, Goethe University Frankfurt, Frankfurt am Main, Germany

**Keywords:** Network models, Object vision

## Abstract

Though the range of invariance in recognition of novel objects is a basic aspect of human vision, its characterization has remained surprisingly elusive. Here we report tolerance to scale and position changes in one-shot learning by measuring recognition accuracy of Korean letters presented in a flash to non-Korean subjects who had no previous experience with Korean letters. We found that humans have significant scale-invariance after only a single exposure to a novel object. The range of translation-invariance is limited, depending on the size and position of presented objects. To understand the underlying brain computation associated with the invariance properties, we compared experimental data with computational modeling results. Our results suggest that to explain invariant recognition of objects by humans, neural network models should explicitly incorporate built-in scale-invariance, by encoding different scale channels as well as eccentricity-dependent representations captured by neurons’ receptive field sizes and sampling density that change with eccentricity. Our psychophysical experiments and related simulations strongly suggest that the human visual system uses a computational strategy that differs in some key aspects from current deep learning architectures, being more data efficient and relying more critically on eye-movements.

## Introduction

Invariance to geometric transformations can be a huge advantage for a visual recognition system. It is important to distinguish between invariance due to the underlying representation, which we refer to as *intrinsic* invariance, and *example-based* invariance for familiar objects that have been previously seen under several different viewpoints. The latter is computationally trivial and is available to any recognition system with sufficient memory and large training data. The first one, which may be hardwired or learned during a developmental period, provides a learning system the ability to learn to recognize objects with a much smaller *sample complexity*, that is with much smaller training sets^1,2^. This is not only a big advantage for any recognition system but it is also a key difference between today’s best deep learning networks and biological vision systems: the most obvious advantage of children versus deep networks is the ability to learn from a (labeled) training set that is several orders of magnitude smaller^3^. The prototypical observation is that we can easily recognize a new object, such as a new face – seen only once – at a different scale.

Current deep networks exploit architectural priors for intrinsic invariance. For instance, Convolutional Neural Networks, which are widely used in computer vision, have an architecture hard-wired for some translation-invariance while they rely heavily on learning through extensive data or data augmentation for invariance to other transformations^4^. Networks that incorporate a larger set of intrinsic invariances, such as rotation-invariance, have been proposed^5–7^. Nevertheless, it is not clear which type of intrinsic invariance should be encoded in more biologically plausible models. As a consequence, it is important to characterize the degree of invariance in human vision, starting from the simplest invariances– scale- and translation-invariance– and evaluate models that reproduce them.

Surprisingly, the available psychophysical results are often incomplete and inconclusive. Most experiments have targeted only translation-invariance, and a review^8^ states that based on experimental data, the role of object position is not well understood and there is little evidence supporting the idea that human object recognition is invariant to position. Findings from previous studies range from “This result suggests that the visual system does not apply a global transposition transformation to the retinal image to compensate for translations”^9^. to “For animal-like shapes, we found complete translation invariance”^10^, and finally to “Our results demonstrate that position invariance, a widely acknowledged property of the human visual system, is limited to specific experimental conditions”^11^. Furthermore little research was conducted on scale-invariance with regard to unfamiliar stimuli (see^12,13^ for studies on scale-invariant recognition of familiar objects. Although a new set of objects different from those in the training phase was tested, the images are still of common objects^13^).

Physiological data on monkeys, on the other hand, give more consistent results on intrinsic invariance in the visual system. A few authors^14,15^ reported that IT responses were invariant to scale and translation, once the monkeys learned a novel object under a single viewpoint. In humans, however, the extent of intrinsic invariant recognition is still unknown (see^16–18^ for studies on primate invariant recognition and^19^ for human invariant recognition of familiar objects).

In the experiments on translation-invariance, it is important to take into account that primate visual acuity depends strongly on eccentricity. Historically the eccentricity-dependence of visual acuity has been studied extensively (see^20^ for a review). In particular, previous studies using letter stimuli^21,22^ found that visual acuity decreases linearly with eccentricity. Therefore, if we consider the range of visual angle in which objects are recognizable for each size, we can define a window of visibility which lower bound is a linear relation between objects’ size and position. The linear relation between recognizable scale and position of an object is also consistent with the physiological data that shows that the size of receptive fields in the primate visual cortex increases with eccentricity^23^. The results imply that fine details, as required for instance to recognize letters at a distance, are visible only to the small receptive fields in the foveola, whereas coarser details, such as those associated with larger letters, are also visible to the larger receptive fields present at greater eccentricities.

The main questions of this paper can thus be phrased as follows. Does a window of invariance exist within the window of visibility? What is its geometry and size? In particular, for visibility there is a linear relation between scale and position. Is the same linear relation also valid for the window of invariance? We investigate these issues by examining human invariant recognition in the one-shot learning scheme, using previously unfamiliar visual stimuli. We also ask whether hierarchical Convolutional Neural Networks can account for the experimental data. In particular, we consider Eccentricity-dependent Neural Networks (ENN). ENNs – described more thoroughly later – implement the hypothesis that the human visual system has hardwired scale-invariance with the size of the receptive fields of the model neurons increasing with eccentricity^2^. These experiments, together with simulations, allow us to characterize invariant recognition arising from intrinsic brain representations.

## Results

**Figure 1:**
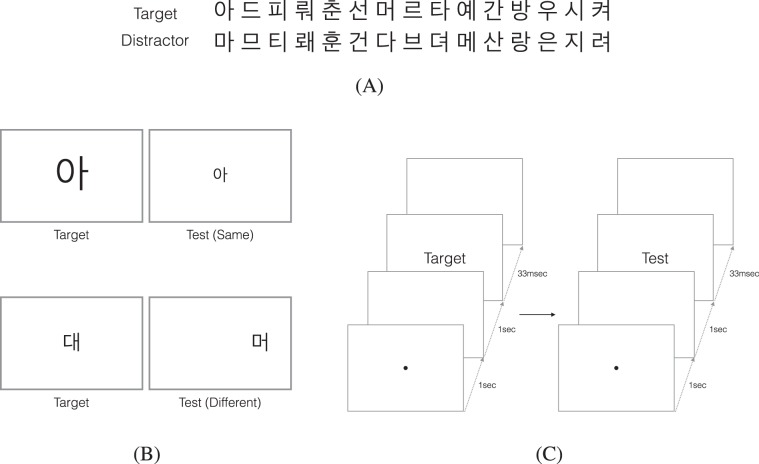
(**A**) Sample stimuli. *Top row*: shows target letters, and *Bottom row*: shows distractor letters paired with the target above. (**B**) Experimental design. *Top*: illustrates a sample trial of scale-invariance experiments, and *Bottom*: illustrates a sample trial of translation-invariance experiments. The test letter was either the same as the target or its pairing distractor letter. (**C**) Experimental procedure. Each target and test letters was presented for 33 msec after a fixation dot was presented for 1 sec at the center of the screen.

To study intrinsic invariance we analyzed results for recognition of unfamiliar letters in one-shot learning. For the one-shot learning task, we flashed a target Korean letter and then a test Korean letter, which was either the same as the target or a different distractor, to non-Korean subjects who were unfamiliar with Korean letters. To investigate invariant recognition to transformations, we varied scale and position of the letters. When testing recognition in the peripheral visual field, we randomized to which side of the visual field letters were presented to prevent that subjects predict the letters’ position, fixate on the stimuli, and observe them with their foveal vision. We limited the presentation time to 33 ms to avoid eye movements. In Fig. 1 we depict the experimental set-up and a set of Korean letters used.

### Experiment 1: Scale-invariance

**Figure 2:**
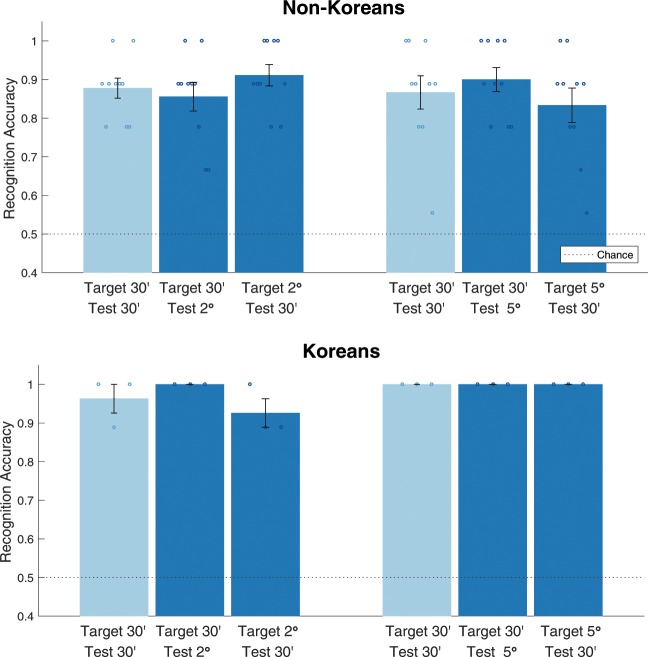
Scale-invariance experimental results. Target and test letters were always shown at the center of the screen, only their size was varied, and subjects responded same or different. Error bars represent standard error (Number of subjects n = 10 for non-Koreans and n = 3 for Koreans conditions).

We tested scale-invariant recognition by flashing both target and test Korean letters at the fixation point in the center of the screen. First, we used 30′ and 2° letter size. In Fig. 2 we compare the three conditions when the size of target and test letters were (30′, 30′), (30′, 2°), and (2°, 30′), respectively, in which the first number of the pair refers to the size of the target letter and the second indicates the size of the test letter. Mean accuracy under all three conditions was higher than 0.85, which is significantly above chance (0.50). Changing the letter size did not have any statistically significant effect on performance ($F(2,18)=0.94,p=0.41$).

We performed a second set of experiments with a greater range of change in scale, in which we tested invariance of recognition with respect to 10-fold increase and decrease of letter size with 30′ and 5° letters. Results were similar to those from the first setting. Mean accuracy was above 0.83, which is significantly higher than chance, and the difference in accuracy among the three presentation conditions was statistically non-significant ($F(2,18)=0.80,p=0.46$).

After observing that visual recognition is robust to scale change in one-shot learning, to compare the range of invariant recognition with that of recognition of familiar objects, we tested native Koreans under the same conditions. The results confirmed that the task was not challenging to Koreans. Mean accuracy for all conditions was above 0.92 (Fig. 2 bottom). When these results were compared with non-Koreans’ data, we did not find any significant interaction between presentation conditions and whether the subjects were Koreans or not (combinations of 30′ and 2° letters: $F(2,22)=0.03,p=0.97$; combinations of 30′ and 5° letters: $F(2,22)=0.23,p=0.80$). We report results using another behavior performance metric *d*′ in Fig. S3, which were consistent with the accuracy results.

### Experiment 2: Translation-invariance

**Figure 3:**
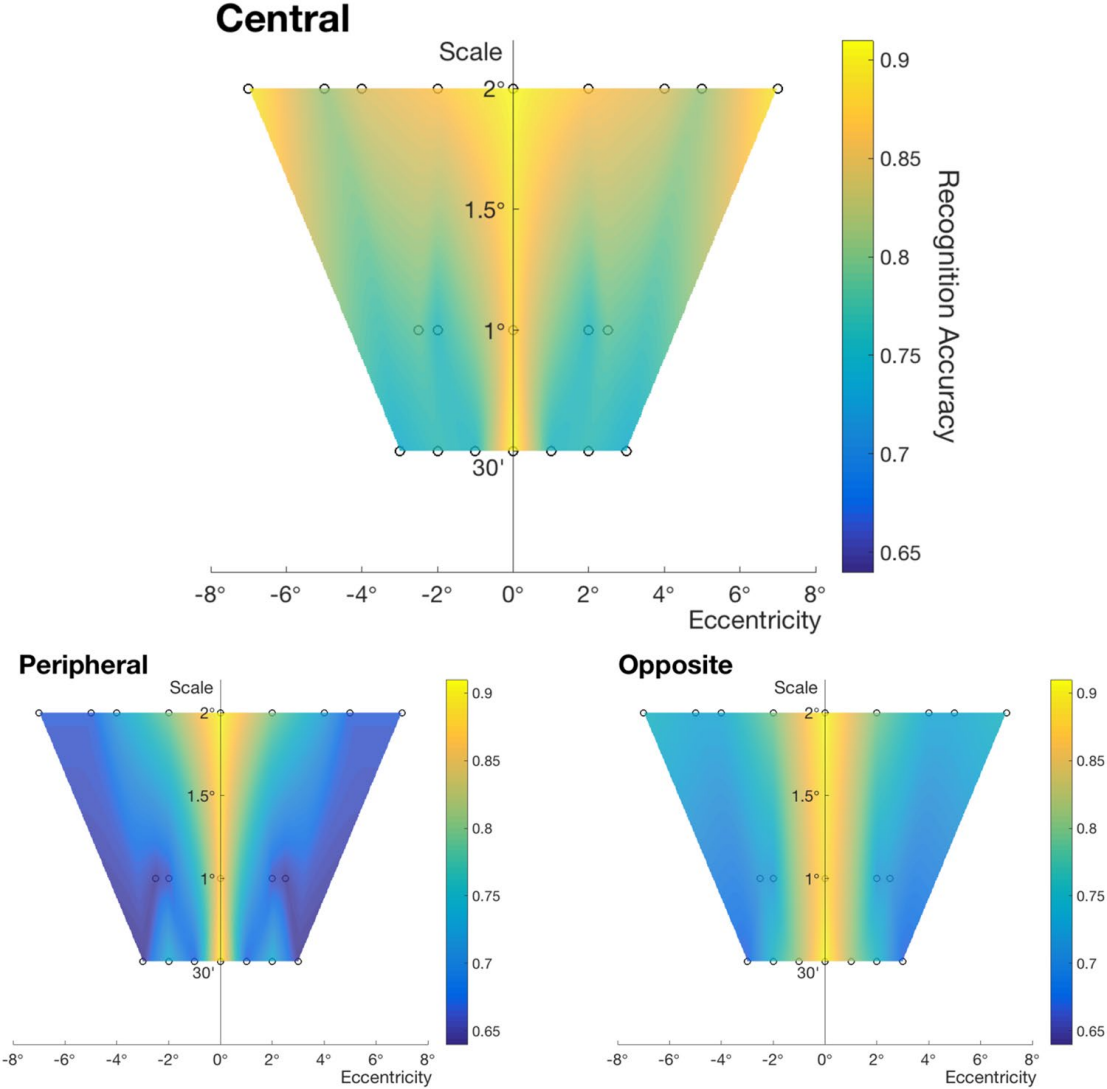
Windows of invariance for different conditions. Recognition accuracy from translation-invariance experiments is shown in a color scale. The central window (*top*) indicates results for learning target letters at the center of the visual field and being tested at another position in the peripheral visual field. Recognition accuracy is shown at corresponding scales and eccentricities of test letters. The peripheral window (*bottom left*) is for the reverse order where target letters are learned in the peripheral visual field and tested at the center. For this condition, as the position of target letters is varied and test letters are fixed at the center, we plot recognition accuracy at the learned scales and eccentricities of target letters. The opposite window (*bottom right*) shows results for learning target letters at a position in the peripheral visual field and being tested at the same distance from the center but in the opposite side of the visual field. In all plots, the tested conditions are marked with circles and other data points are estimated using natural neighbor interpolation (Number of subjects n = 9 for 30′ letter, n = 11 for 1° letter, and n = 10 for 2° letter size conditions).

Next, we investigated translation-invariance by shifting the position of test letters from target letters. We divided the conditions into two categories: learning at the central visual field and learning at the peripheral visual field, based on the position where the target object is learned. We show recognition accuracy at different positions for each scale, which displays the relationship between scale, position, and degree of invariance in Fig. 3 (performance *d*′ is reported in Fig. S4). More details on the experimental set-up are provided in the SI methods section. Recognition accuracy is shown as bar plots in Fig. S1. We also performed similar analyses as for scale-invariance by comparing invariant recognition accuracy with baseline conditions (same position). Unlike scale-invariance experiments, this yielded statistically significant differences in some cases, which suggests limited translation-invariance. We report these results in Fig. S1 and here we further analyze the properties of translation-invariance.

Since in a natural setting, humans are able to observe the unknown objects with their fovea, we first focus on analyzing the central learning condition (Fig. 3 top). For all scales, recognition accuracy was the highest at the center, when there was no displacement, and decreased with increasing distance from the center of the visual field. In addition, the range of translation-invariance increased with the scale of stimuli. While recognition accuracy was maintained high at a position as far as 7° in the periphery for 2° letters, it dropped significantly even at 1° for 30′ letters. Considering the area where recognition accuracy is above a threshold (e.g. 0.85) as the range of invariance, we observed a roughly V-shaped area. We found the same tendency that recognition accuracy depends on eccentricity and scale in peripheral learning conditions.

Additionally, overall recognition accuracy was significantly lower under peripheral learning than under central learning, particularly when there was a change in resolution of test letters from that of target letters (Fig. 3 Peripheral window) i.e. translation-invariance was more limited under peripheral learning. In a related setting with peripheral learning, when target letters are learned in the peripheral visual field and test letters are presented at the same distance from the center but in the opposite side of the visual field, the range of invariance was less limited. Note that under this condition, the resolution of letters did not change and only their position was changed to the opposite side of the visual field. The corresponding window of invariance (Fig. 3 Opposite window) was still more limited than the results from central learning conditions.

#### Does the range of invariance extend with experience?

To compare the properties of intrinsic translation-invariance with those observed in subjects with experience, we tested native Korean subjects with the same experimental set-up as for the above experiments, displayed in Fig. 1 (performance *d*′ is reported in Fig. S5). For Korean subjects, we measured their recognition accuracy using the furthest position tested for each size among the conditions used for non-Korean subjects (30′ letters at eccentricity D = 3°, 1° at D = 2.5°, and 2° at D = 7°). The mean accuracy performance for all three letter sizes was higher than 0.85 (Fig. S2), which confirms that the conditions for which we tested translation-invariance become trivial when the subjects have previous experience.

**Figure 4:**
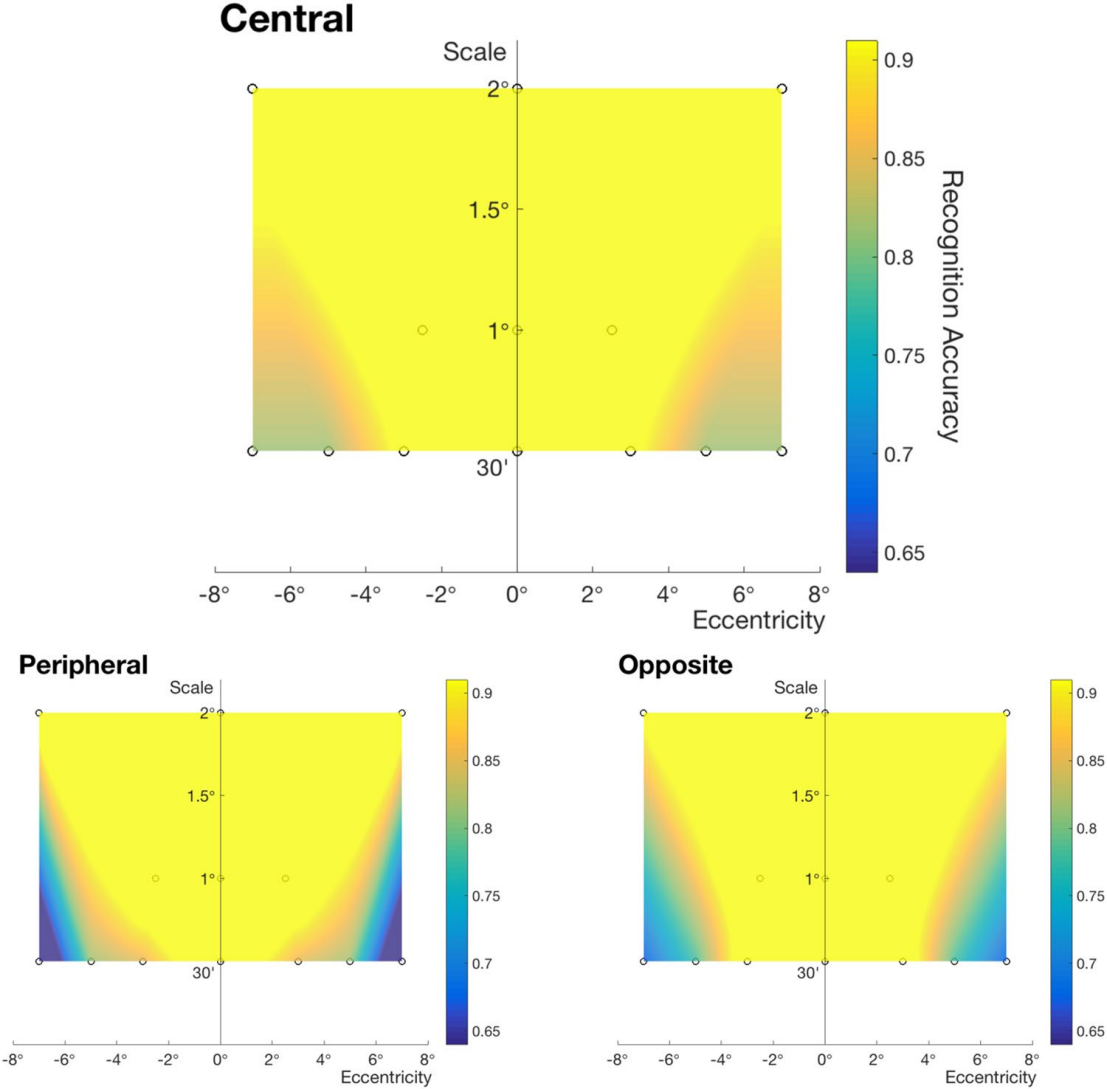
Windows of visibility. They are computed in a similar way as the windows of invariance (Fig. 3), but obtained from testing Korean subjects who are familiar with the visual stimuli (Number of subjects n = 3).

The above set of experiments suggest that the range of recognition is wider when the stimuli are familiar than in one-shot learning, and that recognition performance improved with experience and exposure to the stimuli at different positions. To further investigate the properties and tendency of visibility window, we tested eccentricities D = 5°, 7° for 30′ letters. Compared to non-Koreans’ results, we can confirm that overall recognition accuracy of Korean subjects is higher in Fig. 4. In addition, as in the case of testing non-Korean subjects, the range of visibility window was wider for central learning than for peripheral and opposite learning conditions.

#### Do Deep Neural Networks capture the properties of invariant recognition?

**Figure 5:**
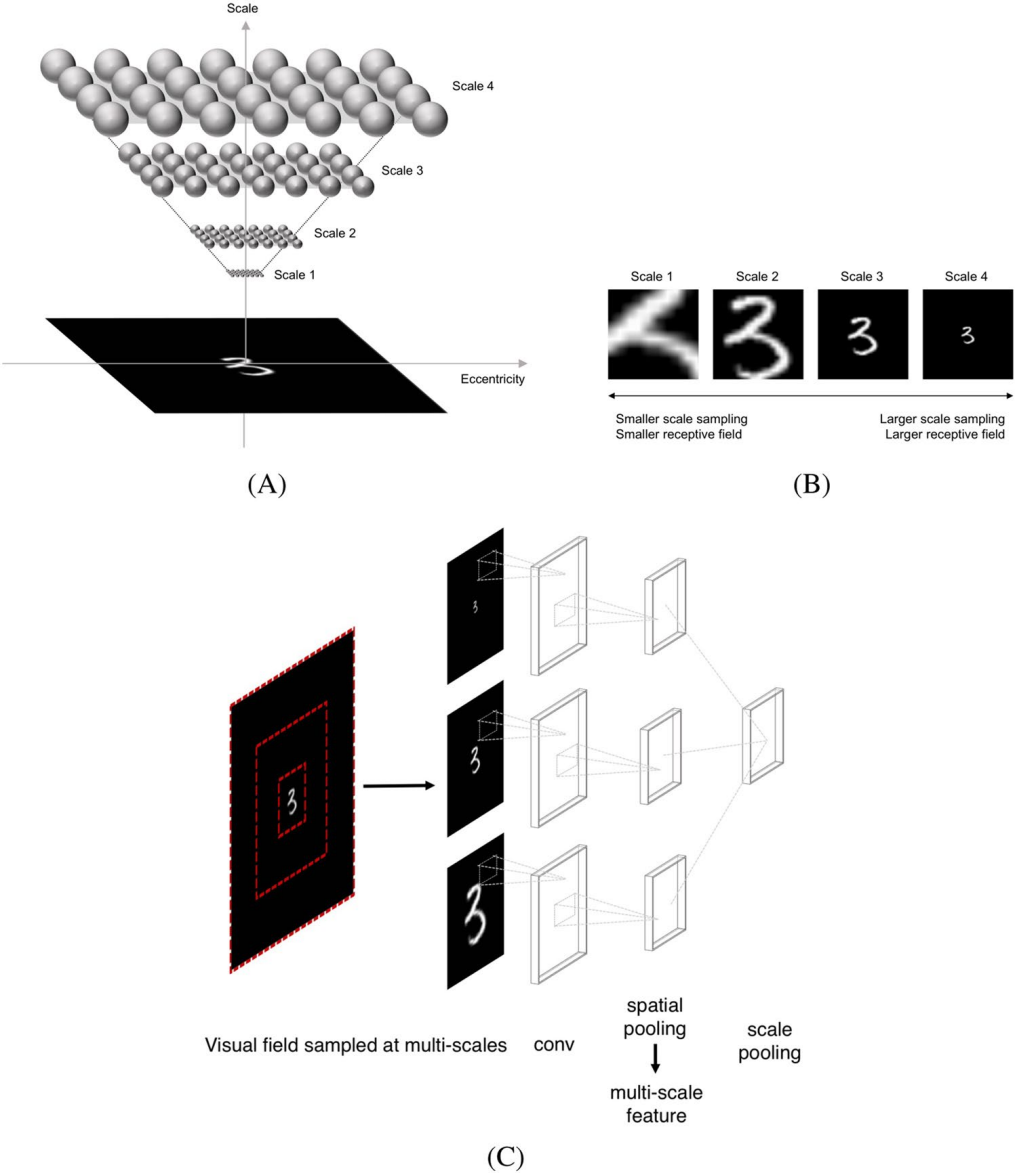
(**A**) Sampling points of the early visual cortex in the plane of eccentricity and scale, both in visual degrees, reproduced from^32^. Each ball represents a neuron, and there is the same number of neurons at all scales. The neurons at a larger scale cover a larger eccentricity than those at a lower scale. (**B**) Multi-scaled centered crops of an input image. The figure shows 4 crops among 10 that are used as the input to Eccentricity-dependent Neural Network. From the left to the right, the scale of input crops becomes larger, which are seen by larger receptive fields. (**C**) Eccentricity-dependent Neural Network. The input to the model is simulated visual field sampled at multiple resolutions as shown in (**B**), and the model is composed of convolutional layers followed by spatial and scale pooling. For simplicity, we visualize a model with one convolutional and pooling layer.

To understand the underlying brain computation that enables human invariant recognition characterized in psychophysical experiments, we compared the experimental data with computational modeling results. In particular, we investigated whether invariance properties observed in human one-shot learning can be learned by examples seen by the model or alternatively, requires an intrinsic architecture for them. We used Convolutional Neural Networks (CNN) to simulate the experimental results, as these models showed a significant success in explaining visual processing in the primate ventral stream^24–27^ and matching behavioral patterns of object recognition with humans^28,29^. A trivial way to achieve invariant recognition, widely adopted in computer vision field, is to use data augmentation to train CNNs^4^. Although models can reach human-level invariant recognition performance for familiar objects with this method, the strategies of CNNs in using diagnostic features were shown to be different from humans^30^. Moreover, it is unknown whether invariant recognition can be transferred to a new category of stimuli, unseen in the training phase. To show the limitation of this example-based invariance in one-shot learning, we compared CNNs with Eccentricity-dependent Neural Networks (ENN)^31–33^. ENNs, depicted in Fig. 5, are modified from CNNs to have scale-invariance built into their architecture and have dependence of receptive field size on eccentricity, consistently with physiology data^23^.

Both CNNs and ENNs were trained on MNIST handwritten digit dataset^34^ with data augmentation of various scales and positions. With this training, the networks should develop top-layer features capable of processing character-like stimuli. Those features are then used to evaluate the similarity of two Korean letters, as in the psychophysics experiments. Two Korean letters are considered to have the same identity if their associated features have Pearson correlation higher than a threshold. Here, we report results from applying a different threshold that maximizes accuracy for each condition. We also included distractor letters in testing so that we evaluate selectivity of the models.

#### Simulation 1: Scale-invariance

**Figure 6:**
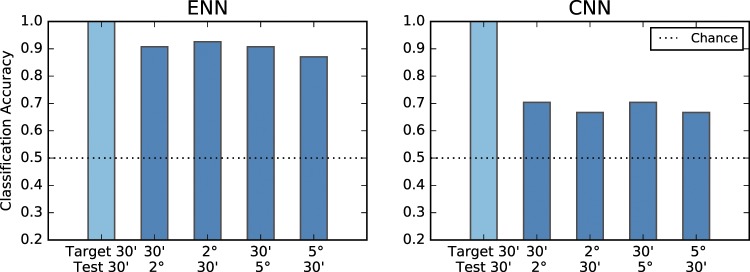
Simulation results on scale-invariance. Scale-invariant representation is assessed by comparing the features of two Korean letters, unseen by models in the training phase, and classifying the letters the same or different, independent from their size. Both ENN and CNN are trained on MNIST handwritten digit dataset^34^ with data augmentation of various scales and shifts. The trained models are used to extract features for Korean letters. Two Korean letters are considered to have the same identity if their associated features have Pearson correlation higher than a threshold. For each condition, we select a threshold that maximizes the classification accuracy.

As described earlier, the psychophysical experiments show that the human visual system is immediately invariant to scale change in one-shot learning. We first tested whether the results with ENNs, which are of course designed to be scale-invariant, fit the data. We evaluated the degree of scale-invariance for Korean letters, which the models did not see during training. As expected, accuracy when the target and test letters are of different size turned out to be significantly higher than chance (Fig. 6 left). Although classification accuracy for testing invariant conditions was lower than that for the baseline condition, when the letter size does not change, this was partly due to the difference between biological systems and computational models. In simulations, since there was no noise, the input images for target and test letters were exactly the same under the baseline condition, which resulted in 1.0 classification accuracy. Overall high accuracy in the testing shows that scale-invariance properties of ENN are consistent with the human data.

We then asked whether a model which has scale-invariance (example-based) for familiar objects shows intrinsic scale-invariance for a new set of objects. To test this hypothesis, we evaluated CNNs for Korean letters. Note that these models were scale-invariant for the trained MNIST dataset. The results obtained with the CNN model (Fig. 6 right) show that classification accuracy when the letter size changes was higher than chance but significantly lower than accuracy for ENN and psychophysical data. This limitation suggests that CNNs with data augmentation cannot account for scale-invariance in one-shot learning.

#### Simulation 2: Translation-invariance

**Figure 7:**
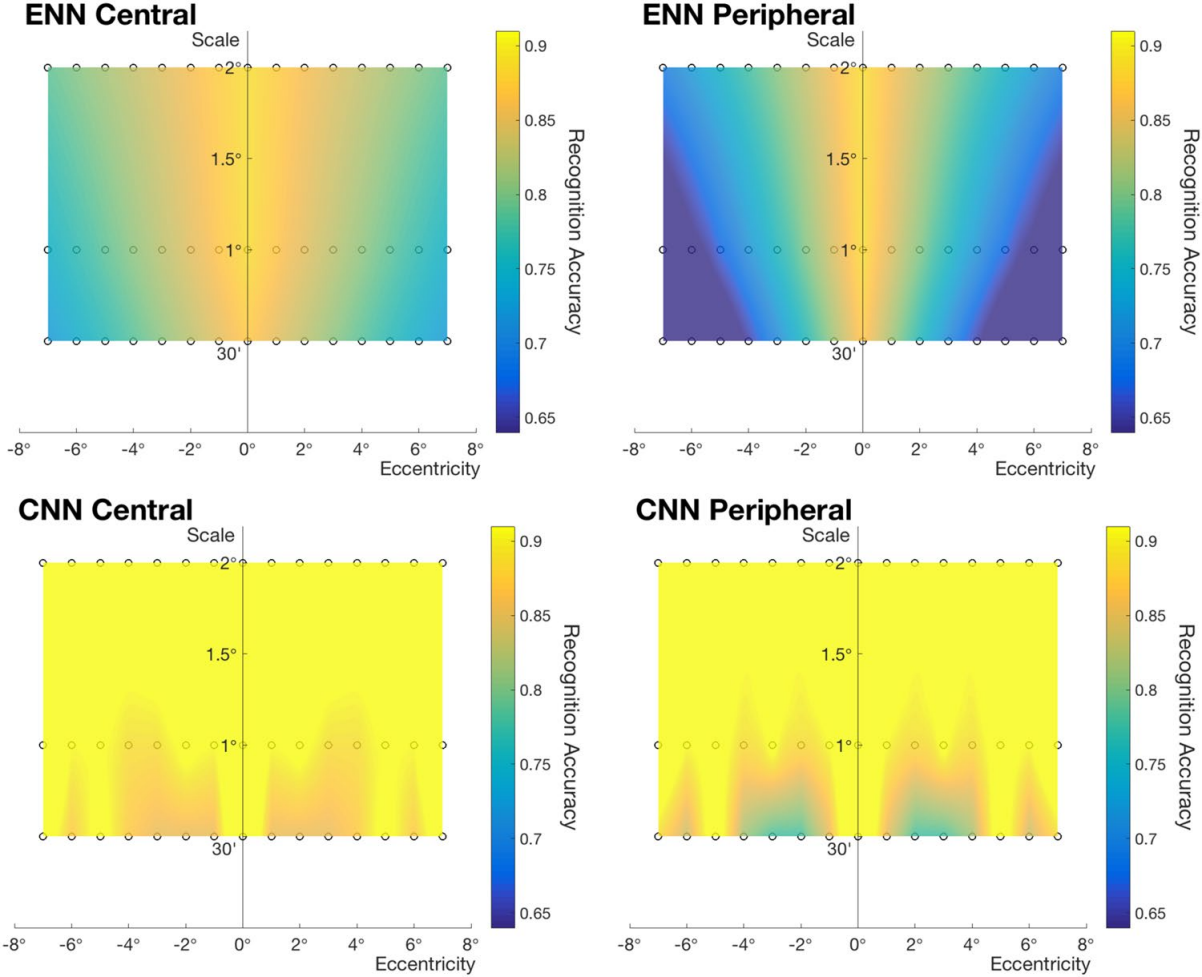
Windows of invariance for ENN and CNN. We test Korean letters in a same-different task, where the position of the letters varies. *Top row*: shows classification accuracy for ENN and *bottom row*: shows that for CNN in an interpolated color scale. For both models, the central window indicates results for using features of target letters placed at the center of the visual field and using features of test letters at a position in the peripheral visual field. The peripheral window shows results for the reverse order of testing. Conditions we tested are marked with circles.

In our psychophysical experiments, the degree of translation-invariance increases with letter size, both under central and peripheral learning. In our simulations (Fig. 7 bottom), CNNs were not able to replicate the property of limited translation-invariance. Accuracy for larger stimuli was higher than that for smaller stimuli, but it did not decrease with eccentricity. These results were expected due to translation-invariant model prior of the CNNs. For ENNs (Fig. 7 top), on the other hand, accuracy decreases with eccentricity while the range of invariant recognition increases with the size of letters, consistently with the psychophysical results. As in psychophysical results, if we choose a threshold classification accuracy and draw an accuracy contour, we can observe a V-shaped area of invariance. (We report raw data in Fig. S7. Window of invariance for ENN (Fig. 7 top) is based on the linear regression of the raw data).

Furthermore, we investigated whether the models can reproduce the asymmetry in recognition rates between central and peripheral learning. The first idea we explored is that the one-shot learning stage stores templates obtained from processing the visual field at multiple scales. Thus, when target letters are presented at the center of the fovea, the associated templates contain all the full range of spatial frequencies. However, when target letters are shown at an eccentricity, since only the central visual field is sampled at high resolution, the templates are effectively low-pass versions of the foveal ones. Therefore, an explanation of the asymmetry between central and peripheral learning may start with the different range of resolutions available for templates memorized in the two situations of foveal vs. “peripheral” learning.

Since CNNs process images at one resolution only, they are unable to account for positional asymmetry in learning, as shown in Fig. 7. In our simulations, features extracted by a CNN by removing the fully connected layer were used to compute Pearson correlation between target and test presentations to evaluate the identity of letters. Thus, there cannot be any asymmetry as a function of the order of the presentations. Within the class of ENN models, we use a network that uses features extracted from all scale channels to “learn” the representation for target letters. In Fig. 5C, these features are the output of convolution and spatial pooling. For the test it uses pooling over the scale channel features. The comparison between stored template and new image is then carried out as follows. The scale channel of the stored letter that has the highest correlation with the test letter is chosen, and then a threshold is applied. The idea is that once we view a target letter with a specific cutoff in spatial frequencies, templates at multiple resolutions lower than that resolution in memory become available: each one can then be compared with the features of the test letter. Clearly the computation of the similarity between target and test is now asymmetric, unlike the original model of using features pooled over different scales and positions for the learning stage^32^ (Results from using the original model are provided in Fig. S8. Scale-invariance simulations using the modified metric are shown in Fig. S6; results are consistent with the conclusion from simulation 1). The results of the simulation showed that accuracy for peripheral learning conditions was lower than that for central learning conditions, similarly to the psychophysical data.

One may consider it may be the case that our method finds the optimal threshold that differentiates letters of the same or different identity, even when the underlying representation between translated objects are actually not being more dissimilar with eccentricity. We additionally confirmed that this is not the case by assessing the raw data, which is Pearson correlation between the same Korean letters at different positions (Fig. S9). The results verified that correlation between the representation becomes lower with eccentricity.

## Discussion

While it is widely agreed that humans are able to process complicated visual information invariant to transformation, so far it remained rather unclear whether this is possible because of previous exposure to the specific visual stimuli at different viewpoints or whether the visual system computes invariant representations for novel objects. To address the issue, we characterized the degree of invariance to transformation in one-shot learning, using stimuli for which the subject had no previous experience. We found that there is significant scale-invariance in recognition. We also found limited translation-invariance that increases with decreasing spatial frequency content of the stimuli, as expected (see for instance^1^). Overall, as a function of eccentricity, the window of invariance is narrower than the window of visibility (i.e. acuity). Further, we observed an asymmetry between learning in the fovea and testing in the periphery with respect to the opposite sequence of training and testing.

Our experimental settings controlling familiarity to the objects as well as position and size of them clarify and extend previous studies on invariant recognition. Previous studies^9–11^ examined invariant recognition to translation when visual stimuli were first learned with the peripheral vision. However, unlike those experimental conditions, humans can freely observe unknown objects, and they mostly use foveal vision for learning target objects, since it is almost exclusively in a laboratory setting that peripheral training may happen. Therefore, our results on the asymmetry between central and peripheral one-shot training suggests a difference between natural and unnatural conditions (we refer as natural condition when the object is centered at the fixation point, and unnatural otherwise). While conclusions from previous studies on very limited position-invariance are drawn from peripheral training condition only, we observed stronger invariant representations in a more natural setting. Also, by testing two subject groups who differed in familiarity to the visual stimuli, we confirmed that invariance depends on familiarity with the visual stimuli, consistent with^10^.

We compared the experimental results with computational models based on neural networks. One of our key contributions is that we conclude that standard CNNs cannot account for these experimental data on invariance, whereas a related class of neural networks, that we call ENNs, can. This suggests that ENNs might be better suited for computationally modeling the visual cortex than CNNs, which have been widely used for modeling the ventral stream^24–27^. Furthermore, our results suggest a rather different computational strategy from the one used in these models. In particular, the limited invariance to eccentrically located targets implies that several quite small “effective images” at different resolutions are available to later visual processing rather than a single large image at a fixed resolution^32,35^. If objects are recognized at multiple resolutions in these effective images – i.e. they are not bound to any specific resolution– the models become scale-invariant.

The limitation of CNNs in contrast with ENNs in explaining scale-invariant recognition highlights the significance of an architectural prior (innate or developed during an early stage of visual experience, see^1,2^). CNNs are designed under the assumption that objects should have the same features regardless of their position (assuming antialiasing is taken care of properly as convolutional architectures designed without considering the classical sampling theorem can also suffer from aliasing effect^36^). For other transformations, it is in principle possible that the models learn an invariant representation through rich training data. They would then be able to extract features invariant to transformations. A theory describing architectures capable of this feature was in fact developed in^1^. We found that, however, invariant recognition in CNNs is highly constrained to the exact type of dataset that are used for training, and there is very limited transfer invariance to other datasets, even when they are similar. This suggests that CNNs mainly develop example-based invariance, limited to a memorized set of data. Our psychophysical results, on the other hand, indicate that human invariant recognition supports an alternative design choice which is consistent with neural networks that enforce scale-invariant representation, as in ENNs.

Although our results support built-in scale-invariance for computational models, the exact implementation details of the ENNs architectures tested here need to be further verified. In particular, pooling all scales at the last layer gives a high degree of scale-invariance, but this may well be different from the operations performed in the visual system. The dynamics of invariant recognition of familiar objects in the human visual system were studied in^19^, and the study suggested that the human visual system develops invariant representation in stages corresponding to different visual areas in the ventral stream. Thus, comparing neural recordings from the ventral stream with different layers in the models will be necessary for refining models that are fully consistent with the brain computation.

Additional future direction of the study would be investigating diagnostic critical spatial frequency in ENNs for object recognition. It was previously observed that critical bands of spatial frequency were scale-dependent except for face images^37^. The critical spatial frequency was measured by testing visual recognition of objects embedded in noise. Though the scale channel selected in ENNs depends on the object size, our results predict that critical frequency is scale-invariant since spatial frequency is normalized by the object size. Due to the different experimental setup, however, it is hard to directly compare our results with previous studies on spatial frequency. In particular, it is unclear how a background of noise would affect the scale channel selection in ENNs. Recognition of such images may involve multiple frequency channels to separate target objects from background. Therefore, analyzing the behavior of ENNs for more complex images will be relevant.

Our work on ENNs have implications for eye-movements. ENNs show greater positional invariance to low-resolution images, which suggests a particular strategy for driving saccades, from low to high frequency channels. Although for each fixation only a small fragment of the input image is processed at high-resolution, information about the peripheral visual area extracted by low-resolution channels enables the models to plan the next saccade towards an informative position in the visual field. In this way an image can be efficiently processed without the need of processing the entire visual field at high-resolution^38^.

The computational strategy of ENNs also implies more robustness to clutter and attention to small parts of an image. It was showed^33^ that a model similar to ENNs does not suffer from crowding at the fovea, regardless of background. On the contrary, CNNs fail to recognize the target if the background at testing is different than the background used at training. This suggests that ENNs in foveal learning condition are able to learn the target object independently of the background, and thus are more robust to clutter. In fact, for detection tasks, where localizing a small target in complex scenes is important, extracting features at multi-scale has proven particularly useful^39^. Due to the nature of the detection networks which are biased to identify only familiar object categories^39–41^, this class of models are not comparable with human psychophysical data obtained from one-shot learning (of course, another discrepancy is that these models do not have resolution decreasing with eccentricity). However, we expect that if the models are modified to learn new additional categories easily, those with explicit multi-scale sampling^39^ would require fewer examples than uniform sampling to learn to detect a new object as in ENNs. Moreover, after some training period of the object, multi-scale channels open up the possibility of selecting the channel that is the most relevant to the contextual information, as suggested by the human behavioral studies by Eckstein *et al*.^42^.

## Methods

### Psychophysical Experiments

#### Stimuli and Setup

To create the stimuli set we used 27 Korean letters as target objects, each of them paired with another Korean letter as distractor, depicted in Fig. 1A. For each trial, a sequence of one of the 27 target letters was shown first as target, followed by the test letter, which is the same letter or its pairing distractor. The letters were black Arial presented in different positions and sizes on a uniform white background in a 60 Hz Dell U2412M monitor. We used the Psychophysics Toolbox^43^ for MATLAB^44^ running on a Linux computer. Subjects were seated at a distance of 1.26 m with a chin rest for stable viewing.

#### Experimental Design

The experimental protocol was approved by the Massachusetts Institute of Technology Committee on the Use of Humans as Experimental Subjects (COUHES), and all experiments were carried out in accordance with the approved guidelines and regulations. Subjects provided informed written consent before the experiment.

Scale-invariance Experiment. To test scale-invariance, both target and test letters were presented at the center of the monitor, and the size of letters was varied. We pursued two blocks of experiments to test invariance to scale in recognition. In the first scale experiment block we tested letter sizes of 30′ and 2°. Specifically, the combinations set of target and test letter sizes were (30′, 30′), (30′, 2°), and (2°, 30′), in which the first element represents the target letter size, and the second the test letter size. Similarly, in the second scale experiment block we used letter sizes of 30′ and 5° with combinations of target and test sizes (30′, 30′), (30′, 5°), and (5°, 30′), respectively. The same group of subjects participated in both blocks of scale experiments, with at least a day apart to ensure that the subjects did not remember the stimuli set.

Translation-invariance Experiment. Translation-invariant recognition was evaluated by keeping the size of target and test letters constant and changing the position of test letters with respect to the position of target letters. We divide the tested conditions into two categories:

1. Learning in central vision, where target letters were presented at the subject’s visual fixation point, which was in center of the monitor. In this condition, test letters were presented in the same position as the target (represented as (0 → 0)) or at the subject’s visual periphery. We indicate the latter as (0 → D), in which 0 is the target position at the center of the screen, and D indicates the eccentricity in visual degrees of the test letter position from the fixation point.
2. Learning in peripheral vision, where target letters were presented at the subject’s visual periphery. Then, the test letter appeared at the same eccentricity as the target letter (represented as (D → D)), at the center, (D → 0), or at the opposite side with the same eccentricity as the target letter, represented as (D → Opp).

We tested both conditions of central and peripheral vision with: i) eccentricities D = 1, 2, 3° with constant letter size of 30′, ii) eccentricities D = 2, 2.5° with letter size of 1°, iii) eccentricities D = 2, 4, 5, 7° with letter size of 2°. We tested larger letters for a wider range of displacement to reflect that the range of visibility increases linearly with the letter size^21^.

Since translation-invariance experiments had more conditions than scale-invariance experiments, and the same set of 27 Korean letters was used, the set was repeated in two separate sessions. First, subjects were tested on 27 trials and instructed to come back for the second session after taking a break of at least 40 minutes, to ensure that they did not remember the letters.

Also, we designed translation-invariance experiments such that the same group of subjects participated in two or three eccentricities of displacement for the same letter size, again with at least a day apart between two displacement conditions. The repetition was limited to three times to prevent subjects from developing familiarity with the stimuli, while enabling us to isolate the effect of displacement on the degree of invariance from subjects’ individual difference. Specifically, the same group of subjects participated in all conditions for 30′ letter size, and another group in all conditions for 1° letter size. For 2° letters, the same subject group was tested for D = 2° and 7°, and another group for D = 4° and 5°. The subjects that participated in translation-invariance experiments were different from the group participated in the scale-invariance experiments.

#### Participants

In order to examine the degree of invariance in a one-shot learning task, it is crucial that the stimuli were novel objects to subjects. We recruited participants in the experiments who were not familiar with Korean letters. All subjects had normal or corrected-to-normal vision. We tested 10 subjects for the scale-invariance experiments, and between 11 and 12 subjects for the translation-invariance experiments (for 30′ letter conditions: 12 subjects, 1° letter conditions: 11 subjects, 2° letter conditions for D = {2°, 7°} and D = {4°, 5°}: 12 and 11 subjects, respectively). If a subject performed worse than 0.6 accuracy performance for the trivial condition, where target and test letters were the same size presented at the center, (0 → 0), the subject was excluded from further analyses. Since the same group of subjects participated in two or three displacement conditions for comparison, if a subject performed below the baseline criteria for one displacement condition, the subject was excluded from other displacement conditions as well. After excluding the subjects below the baseline criteria, for scale-invariance experiments, 10 subjects were included. For translation-invariance experiments, 9 subjects per condition were included for 30′ letter conditions, 11 subjects per condition for 1° letter size, and 10 subjects per condition for 2° letter size.

We also tested 3 Korean subjects to confirm that the designed task is trivial and find the range of visibility window for subjects who have prior experience and memory of Korean letters. Note that for Koreans, we used the same experimental setup and task; yet, it was not testing invariant object recognition in one-shot learning, but visibility of the letters in different sizes and positions.

#### General Experimental Procedure

Accuracy for recognizing letters was measured in a same-different task. Subjects were instructed to first fixate a black dot at the center of the screen. After 1 sec, the fixation dot disappeared and a target letter was presented for 33 msec, followed by a white screen for 1 sec. Then, the fixation dot reappeared for 1 sec, followed by a test letter for 33 msec, again followed by a white screen for 1 sec. Finally, the question of the task appeared, in which the subject was asked if the target and test letters displayed previously were the same or different. In Fig. 1C a sample sequence of letter presentations is shown. Every trial was composed of new letter pairs, and randomly choosing if the test letter was the same as the target or the distractor. The presentation time was limited to 33 msec to avoid eye movements, which ensured that the subjects would view the letters at the designed eccentricity.

In both scale- and translation-invariance experiments, the order of stimuli was randomized. The number of same and different trials as well as presentation on the left and right visual field was balanced. Each condition had the same number of trials.

### Model Experiments

To contrast the human behavioral data on invariance with computational modeling results, we evaluate Eccentricity-dependent Neural Network (ENN), which was proposed by^32^ and previously studied in^31,33^. In particular, we demonstrate that ENN is robust to change in scale, and validate that it captures the major characteristics of translation-invariance observed from human experimental data. We test a Convolutional Neural Network (CNN) as a control to show that invariance properties of ENN, especially scale-invariant representation of novel stimuli, are derived from the architectural design of the model rather than a consequence of training with multiple scales and positions.

#### Models

Eccentricity-dependent Neural Network (ENN). ENN (depicted in Fig. 5) builds on two key properties of retinal sampling^32^. One is that there are receptive fields of different sizes for a specific position^45^, and the other one is that the size of receptive fields for each position increases with eccentricity^23^. The model achieves invariance through weight-sharing and pooling across different positions and scale channels. As we hypothesized that the model captures invariant representations to transformations, we tested this model for the comparison with behavioral data on invariant object recognition.

On the implementation level, ENN is based on a CNN. The primary difference between ENN and CNN is that the input to ENN is multi-scaled centered crops of the input images. Figure 5B shows an example set of multi-scaled crops of input images. This way, the center of an image, which corresponds to the foveal region, is sampled at multiple resolutions. The peripheral part of an image is sampled only at a low resolution. Different scale channels have shared weights and in addition to spatial pooling, the model has pooling over different scales. For the results of simulations we partly used the implementation provided by^33^.

ENN that we tested has four layers and a fully connected layer at the end, resembling V1-V2-V4-IT-PFC in the human ventral stream. The size of stimuli or receptive fields are measured in pixels, so we introduced a hyperparameter for the conversion between number of pixels and visual angle, which is 450 pixels to 1°. With this correspondence, we could compare modeling results with human data more directly. For instance, to extract features of 30′ letters, we placed letters of size 225 pixels in the simulated visual field for the model. As discussed previously, the input to the model is multi-scaled centered crops of images, and we use 10 crops, increased in size exponentially by a factor of 1.5. The entire visual field processed by the model is approximately 19°.

We tested different convolutional and pooling schemes over space and scale, and here we have reported the one that matched human behavioral data most closely. The first layer has a kernel size of 11 × 11 pixels convolution with a stride of 4 pixels and 5 × 5 pixels spatial pooling with a stride of 2 pixels. Other layers have a convolutional kernel size of 5 × 5 pixels with a stride of 1 pixel and a pooling kernel size of 5 × 5 pixels with a stride of 2 pixels. When scale-pooling was used on top of spatially pooled features i.e. to explain scale-invariance or to extract features of the test letters, 10 scale channels were max-pooled at the last layer.

When choosing parameters of the network, we confirmed that ENN and human pyschophysical data empirically matched by comparing the window of visibility for digit recognition. For 30′ digits, it was measured that at around 10° from the center of the fovea, recognition accuracy was 67% for humans^22^. If we do a linear interpolation for approximation, accuracy would be about 77% at around 7° for the same size of digits. Using our conversion ratio between pixels and visual angle, we observed accuracy of 72% for 30′ MNIST digits at 7° for ENN, roughly matching the human accuracy. This conversion ratio together with the parameters in the network are also consistent with the theoretically estimated size of the smallest receptive fields^46^.

Convolutional Neural Network (CNN). The parameters used in CNN were the same as ENN, except that there was no multi-crop input channels or pooling over scales, since the model had only one scale channel. The resolution of the input to the model was chosen such that it matched that of the 5th scale channel in ENN, which is its mid-resolution.

#### Statistical Analysis

No statistical methods were used to predetermine sample sizes (number of subjects), but our sample sizes are similar to those reported in previous studies using similar experimental procedures (studies testing recognition of familiar letter stimuli^21,22,47^ and testing invariant recognition of objects^10,13^). We analyzed the percentage of correct responses, combining both same and different trials. For all parametric tests, data distribution was assumed to be normal, but this was not formally tested. To analyze the difference in mean accuracy among three or more conditions, we computed analyses of variance (ANOVAs) or repeated measures ANOVAs, depending upon whether the data were acquired from different group of subjects or the same groups, respectively. Correlation between features in simulations was Pearson’s *r*.

## Supplementary information

Scale and translation-invariance for novel objects in human vision.

## Data Availability

The data and the code supporting the findings of this study are available from the corresponding author upon reasonable request.
