# Supplementary material for: Scale and translation-invariance for novel objects in human vision
Source: Sci Rep. 2020 Jan 29;10:1411. doi: 10.1038/s41598-019-57261-6 (PMC6989457; doi:10.1038/s41598-019-57261-6)
Supplement: Supplementary file 1 — Scale and translation-invariance for novel objects in human vision. [file 41598_2019_57261_MOESM1_ESM.pdf]

Scale and translation-invariance for novel objects in human vision

Yena Han Gemma Roig Gad Geiger Tomaso Poggio

Supplementary Information

SI Experiment 2: Translation-invariance experimental results

We tested two categories for learning: learning at the central visual field and learning at the peripheral visual field, based on the position where the target object is learned. When we compared mean accuracy for a condition with another condition tested in the same subject group, we applied two-tailed paired t-tests. Results are shown in Figure S1. The same results are visualized in a color scale in Figure 3.

Learning in the Central Visual Field For the specific presentation conditions, we use the notation (0→D), where 0 represents that target letters are shown at 0°, which is the fixation point, and D indicates the eccentricity in the peripheral visual field at which test letters are presented. The range of D was varied depending on the letter size.

Accuracy for (0→D) When target letters were presented at the center and test letters at the periphery, accuracy for every combination of size and position was lower than the baseline condition (0→0). Statistically, this difference between (0→D) with (0→0) was significant for 30′ letter size presented at eccentricity D = $\{1°, 3°\}$, and 1° letters with D = 2° ($t(8) = 2.31, p = 0.05$; $t(8) = 3.54, p = 0.01$; $t(10) = 3.32, p = 0.01$). To test whether limited translation invariant recognition is mainly due to the lack of transferability between two positions or noisy representation of peripheral vision, we examined accuracy for (D→D), which represents the

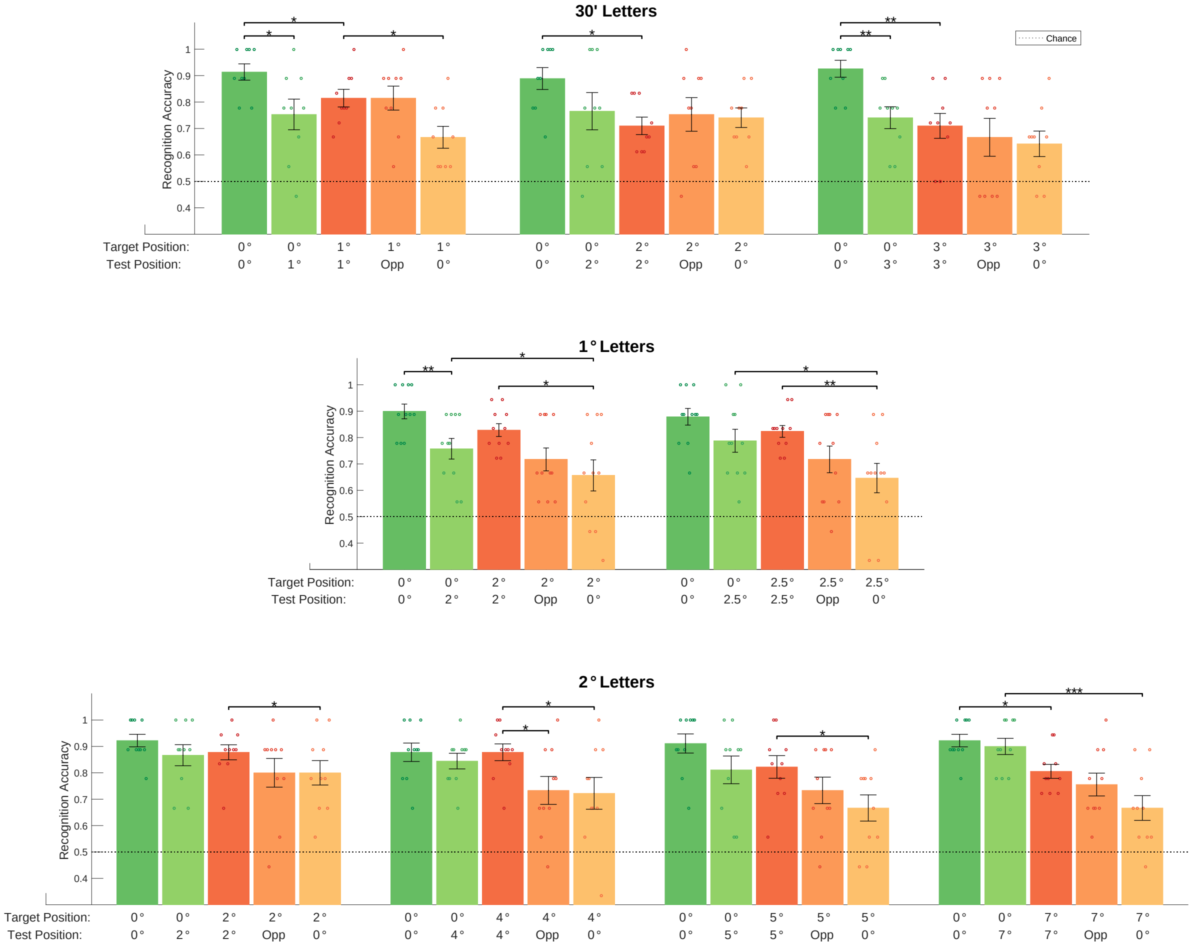

Figure S1: Translation-invariance experimental results for non-Koreans. Three letter sizes (30′, 1°, 2°) were tested and the range of translation was varied depending on the letter size. For each letter size and displacement, 5 different testing conditions, where target and test letters were shown at different positions, are plotted. Target and test letter positions are indicated below each bar. Opp represents testing at the opposite of the visual field in the same eccentricity as the target letter. Error bars represent standard error (Number of subjects n = 9 for 30′ letter, n = 11 for 1° letter, and n = 10 for 2° letter size conditions. $^{*}p < 0.05, ^{**}p < 0.01, ^{***}p < 0.001$ two-tailed paired t-test).

limit on acuity in one-shot learning. The difference between the conditions (0→D) and (D→D) was not significant in any of the conditions (all ps > 0.05). Moreover, we observed lower accuracy for the (0→D) condition for 30′ letter size than for 1° or 2° letters, where there was more limit on acuity, which indicated that imperfect translation-invariant recognition for (0→D) may be attributable to lower acuity. We consider the effect of lower acuity in one-shot learning and transferability to another position altogether as the factors of limited translation-invariance since we confirmed that Koreans did not have limitation on both factors under the same testing conditions (Figure S2).

Learning at the Peripheral Visual Field

To further explore the extent of translation-invariance in the limited acuity conditions in one-shot learning, we next investigated the conditions when target letters are learned in the peripheral visual field. These include conditions (i) (D→0), in which the target letter is presented at an eccentricity of D°, and the test letter at the fixation point; (ii) (D→Opp) in which the target letter is learned at eccentricity D°, and test letter is presented at the same eccentricity but in the opposite site of the visual field, and the baseline (D→D) in which both target and test letters are presented in the same position.

Accuracy for (D→0) We observed that recognizing test letters invariant to position was limited when target letters were learned at the periphery and test letters were presented at the fixation point. Accuracy for (D→0) condition was lower than that for the control condition (D→D) for 1° and 2° letter sizes. Statistical evaluation also confirmed that the difference was statistically significant ($p < 0.05$ in all cases, except for 2° letters at D = 7°, where $p < 0.06$; specifically, for 1°letters at D = $\{1,\ 2°\}$: $t(10) = 3.00, 3.25, p = 0.01, 0.01$, respectively; for 2°letters at D = $\{2,\ 4,\ 5,\ 7°\}$: $t(9) = 2.49, 2.32, 2.54, 2.20$, $p = 0.03, 0.05, 0.03, 0.06$, respectively). However, we did not observe the same effect for 30′ letter size, for which the accuracy for (D→0) condition was lower than (D→D) for D = $\{1°,\ 2°,\ 3°\}$, but only for D =

1° was the difference statistically significant ($t(8) = 3.2, p = 0.01$). This different behavior for small letters ($30'$) than larger letters ($1^\circ$and 2°) might be due to the fact that larger letters have a mean recognition accuracy for the baseline condition, (D→D), higher than 0.80. On the other hand, the mean accuracy for $30'$ letter size for (D→D) with D = $\{2^\circ, 3^\circ\}$ was much lower than 0.80 (both 0.71). This suggests that recognition for $30'$ letter size at the periphery in the same eccentricity for both target and test letters is limited by acuity in one-shot learning. The small difference with respect to (D→0) might be due to such limited acuity. Thus, unless there was a pronounced limit on visual acuity at a particular position in one-shot learning, causing accuracy for (D→D) to be about or lower than 0.80, we observed significantly limited translation-invariant recognition.

Accuracy for (D→Opp) We next sought to test invariant recognition when the position of letters change, but not their resolution. To evaluate this condition, we tested the condition (D→Opp), where letters were translated to the opposite side of the visual field while preserving their distance from the central fixation point. Since both target and test letters were presented in the peripheral visual field for (D→Opp), accuracy could be limited by the acuity in one-shot learning, corresponding to the tested position. Thus, we compared accuracy for (D→Opp) with that for (D→D) to find whether there was a loss in recognition due to displacement in addition to the limit on acuity in one-shot learning. For $30'$ letter size, mean accuracy for (D→Opp) was slightly higher than (D→D) for D = $\{1^\circ, 2^\circ\}$ and lower than (D→D) for D = 3°, yet none of them was statistically significantly different (all ps > 0.5). For 1° and 2° letter size, the mean accuracy for (D→Opp) was lower than the baseline condition (D→D). However, except for the setting of 2° letter size with D = 4° ($t(9) = 2.49, p = 0.03$), none of them resulted in a statistically significant drop in accuracy (all ps > 0.05). These results suggest that acuity has a more significant effect on recognizing $30'$ letters than larger letters for (D→Opp) condition. Discriminating 1° or 2° letters had a marginal performance loss due to displacement to the

opposite side of the visual field.

Asymmetry between Central and Peripheral Learning

One notable result is that the order for which condition letters were learned first had an effect in invariant recognition. For every size and position of letters, the mean accuracy for (0→D) was higher than that for (D→0). In statistical evaluation, 1° letters with D = 2° and 2.5°, and 2° letters with D = 7° had a statistically significant difference ($t(10) = 2.47, p = 0.03$; $t(10) = 2.97, p = 0.01$; $t(9) = 5.55, p = 0.0003$, respectively). As argued above, this might be because the acuity in one-shot learning limits the learnability to the level of accuracy at (D→D).

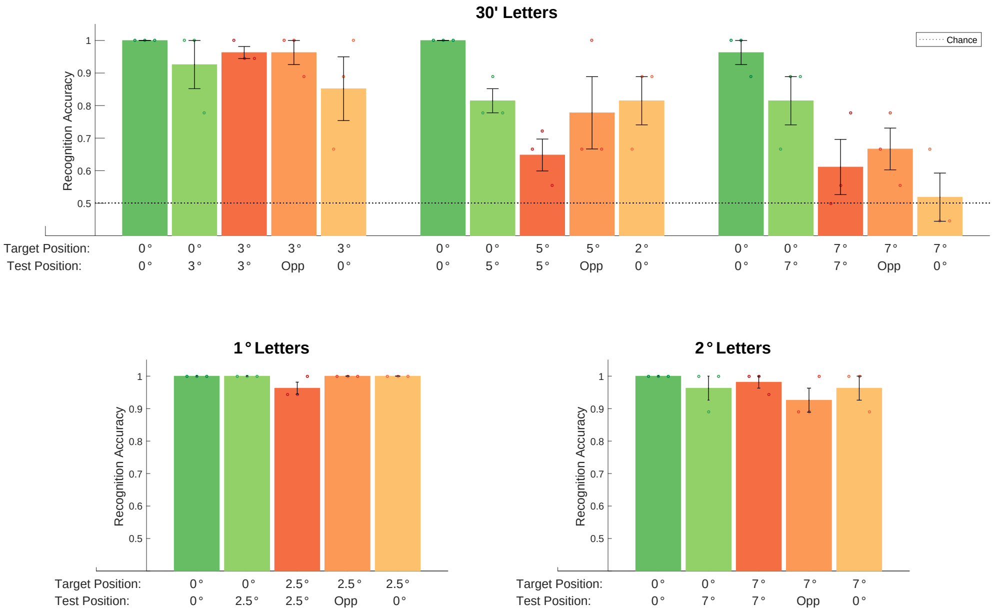

Figure S2: Translation-invariance experimental results for Koreans. We tested the positions with the largest eccentricity among the conditions we tested non-Korean subjects for each size of letter (30′ letters at D = 3°, 1° at D = 2.5°, and 2° at D = 7°). In addition, to investigate the range of visibility window, recognition accuracy for 30′ letters at D = 5° and 7° is plotted. Error bars represent standard error (Number of subjects n = 3 for each condition).

SI: Psychophysical results using sensitivity index d'

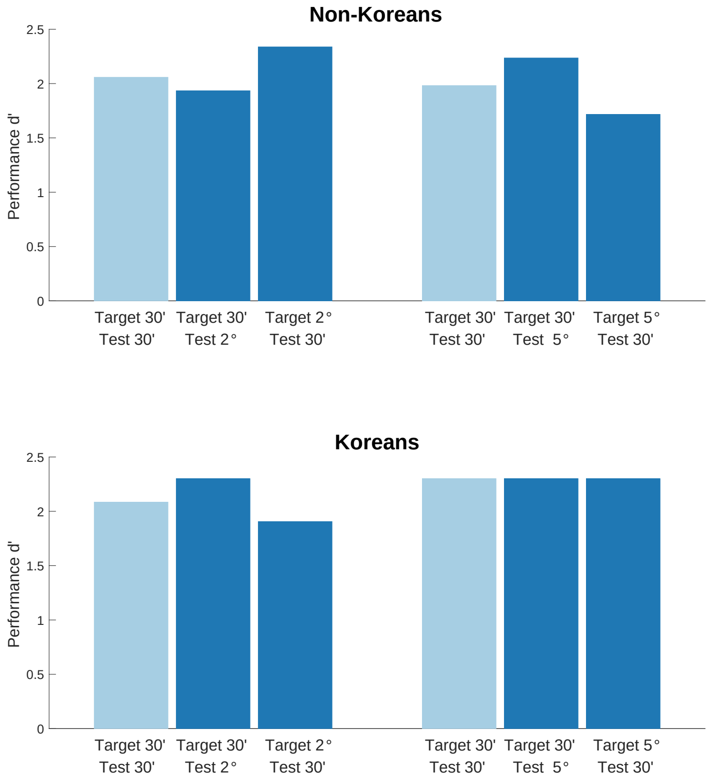

Figure S3: Scale-invariance experimental results, showing d'. We computed d' = Z(Hit) - Z(False alarm), where Z is the inverse of the cumulative Gaussian distribution. Hit and false alarm rates were the average across all subjects' data (n = 10). To bound the values of d', we added 0.5 to both hit and false alarm rates and 1 to both the number of signal trials and the number of noise trials [1]. The overall results showing robustness to scaling is consistent with Figure 2.

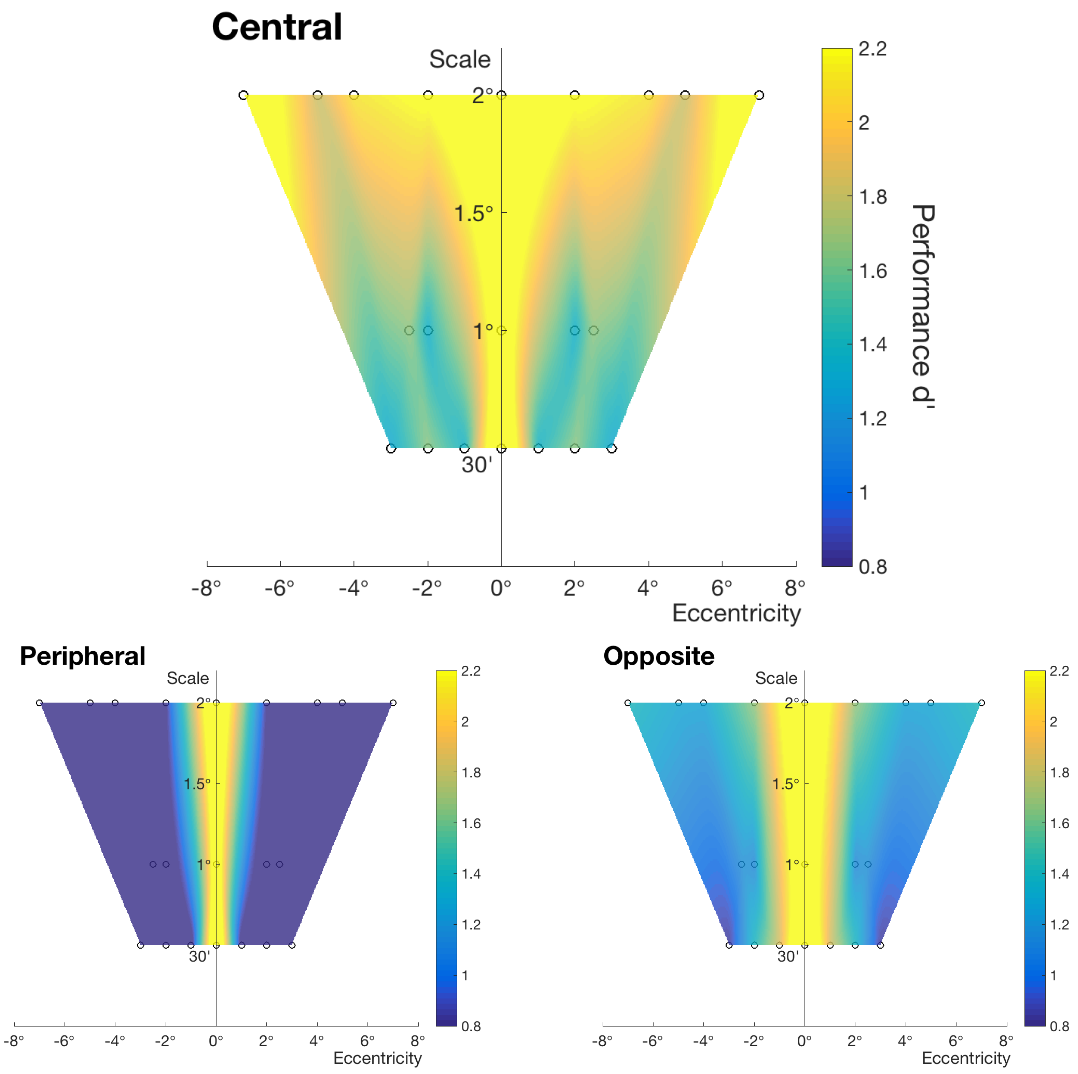

Figure S4: Windows of invariance, showing d'. As in Figure 3, we show d′ for three different conditions (central, peripheral and opposite learning). Consistent with Figure 3, we find that the range of invariance increases with scale for the central learning condition. Also, peripheral and opposite learning conditions result in a narrower range of invariance than the central learning condition.

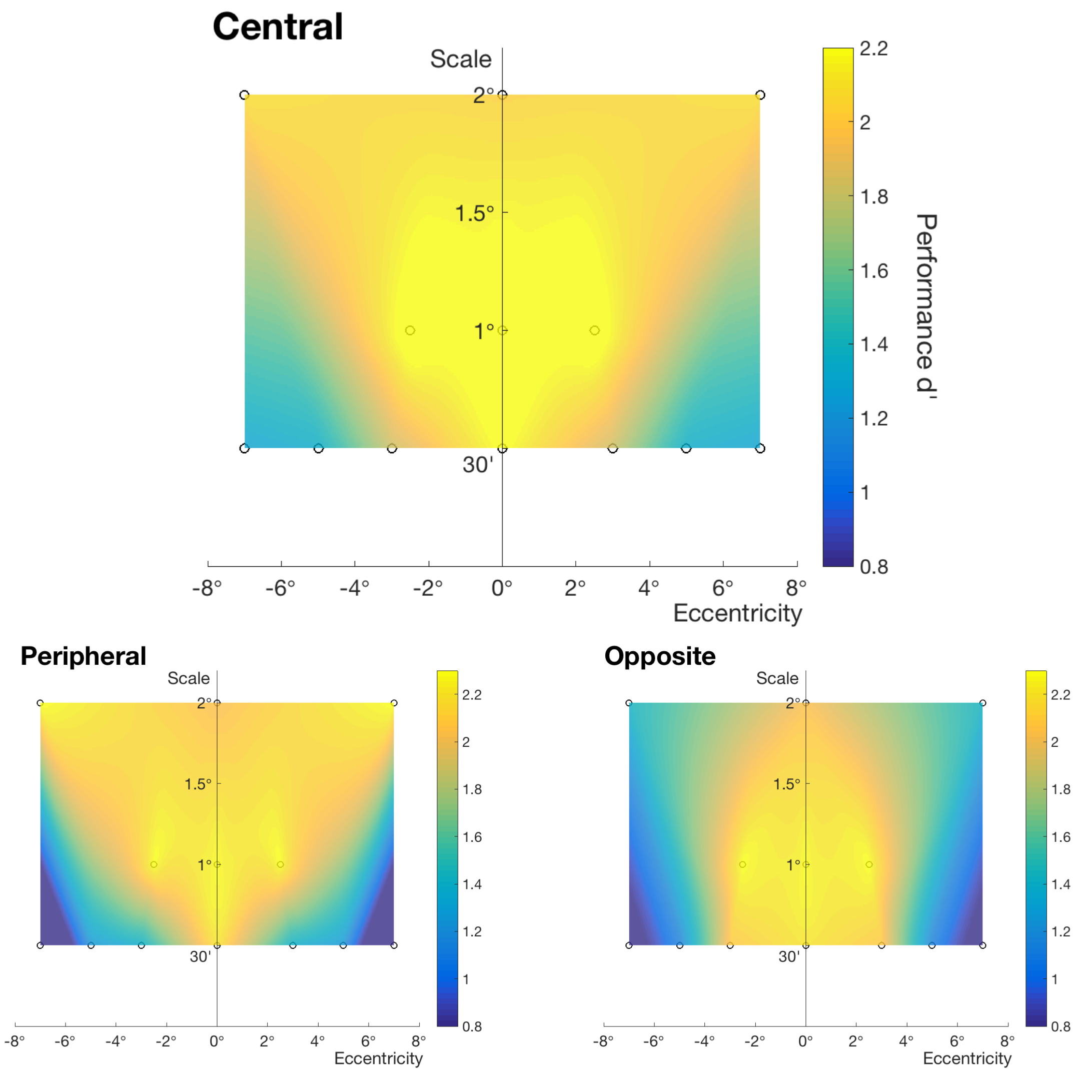

Figure S5: Windows of visibility, showing d'. As in Figure 4, we show that the range of invariance is extended with experience. Overall results are consistent with Figure 4, except unlike in Figure 4, we observe a narrower range for 2° letters than that for smaller letters for some contours of d' values (e.g. $d' = 2$). However, for smaller d' values, this is not the case. Also, the results are consistent with our main conclusion that the window of visibility is wider than the window of invariance.

SI: Scale-invariance supplementary results for ENN

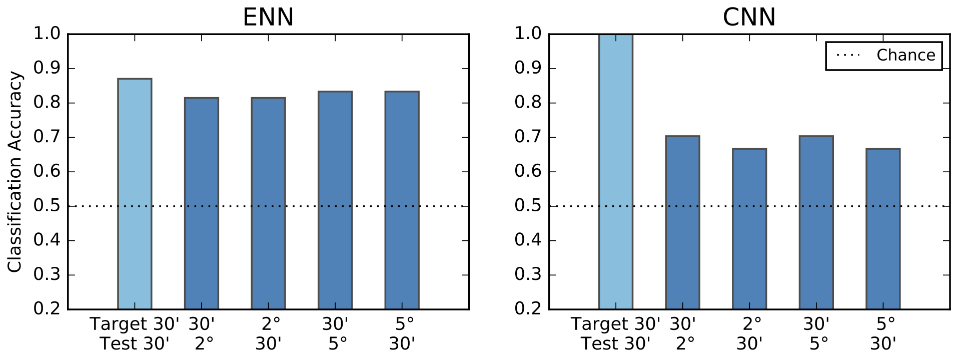

Figure S6: Simulation results on scale-invariance using multi-scale features. Testing conditions are identical to those for Figure 6. Instead of using max-pooled features, we use features from all scale channels for target letters. See the results section.

SI: Translation-invariance supplementary results for ENN

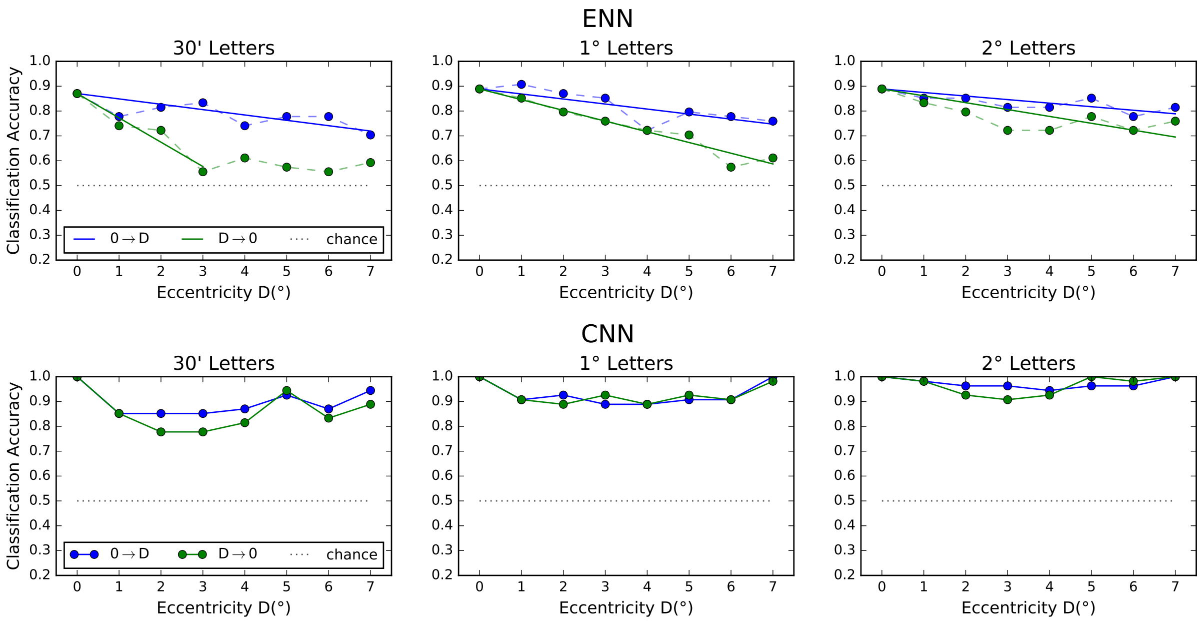

Figure S7: Simulation results for translation-invariance. (0→D) indicates the central learning conditions, when target letters are placed at the center of the visual field and test letters at an eccentricity D° in the peripheral visual field. (D→0) indicates the reverse order of testing which is the peripheral learning conditions. To reproduce asymmetry in recognition rates between central and peripheral learning ENN templates associated with target letters are from all scales. Each scale is matched with the features from test letters, which are pooled over scale channels, to find a scale that maximizes the correlation. A linear fit is plotted for ENN.

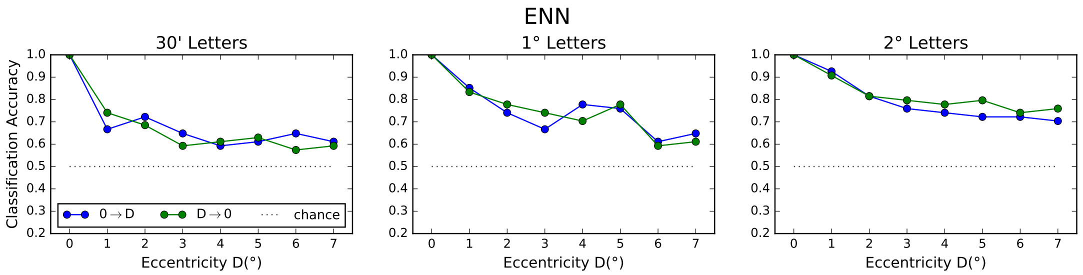

Figure S8: Translation-invariance simulation results from using features pooled over all scales and positions. This method has limitation to explain the asymmetric recognition accuracy between central and peripheral learning. In the main text, we instead use features from all scales to reproduce translation-invariance experimental results. See the results section.

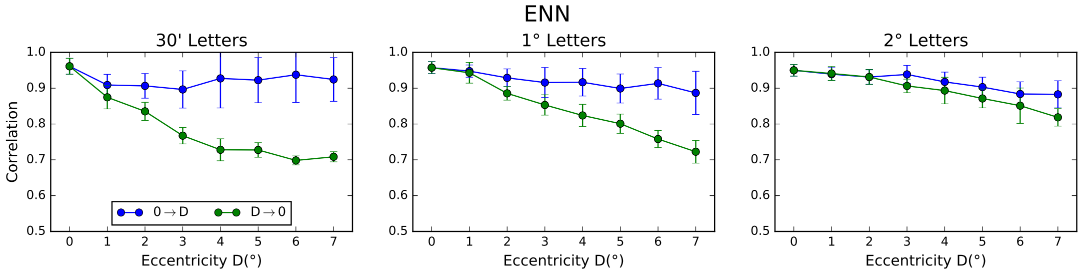

Figure S9: Pearson correlation between same Korean letters at different positions. See the results section. Error bars represent standard deviation (Number of letters n = 27).

References

[1] M. J. Hautus. Corrections for extreme proportions and their biasing effects on estimated values of d'. *Behavior Research Methods, Instruments, & Computers*, 27(1):46–51, 1995.
